# Response to: Comment on “Detecting Key Genes Regulated by miRNAs in Dysfunctional Crosstalk Pathway of Myasthenia Gravis”

**DOI:** 10.1155/2017/5359434

**Published:** 2017-08-14

**Authors:** Yuze Cao, Jianjian Wang, Huixue Zhang, Qinghua Tian, Lixia Chen, Shangwei Ning, Peifang Liu, Xuesong Sun, Xiaoyu Lu, Chang Song, Shuai Zhang, Bo Xiao, Lihua Wang

**Affiliations:** ^1^Department of Neurology, The Second Affiliated Hospital, Harbin Medical University, Harbin, Heilongjiang 150081, China; ^2^Department of Neurology, Xiangya Hospital, Central South University, Changsha, Hunan 410008, China; ^3^College of Bioinformatics Science and Technology, Harbin Medical University, Harbin, Heilongjiang 150081, China

We would like to thank the editor for providing valuable comments. We also thank Dr. Panse for the comments [1]. The comments and contributions of Dr. Panse are very precious and add value to the paper. We would like to reply to the comments on our manuscript titled “Detecting Key Genes Regulated by miRNAs in Dysfunctional Crosstalk Pathway of Myasthenia Gravis” [2]. We have tried our best to reply to the comments by providing our opinions below. Meanwhile, we included the corrections to the article in corrigendum [3].

We rechecked our manuscript about the two different “tissues” carefully. To overcome this issue, we applied pathway filter analyses instead of using miRNA-mRNA regulatory pairs directly. In our paper, KEGG pathway enrichment analyses were performed for predicted targets of miRNA and mRNA, respectively. Then, pathways enriched for differentially expressed mRNAs were used to filter predicted targets to identify dysfunctional pathways in myasthenia gravis (MG). Interestingly, significant crosstalk was detected between five of these pathways based on a cumulative hypergeometric distribution. Furthermore, we detected some key genes mediated crosstalk between dysfunctional pathways, and these genes were regulated by MG-related miRNAs. Besides, we also reviewed more literatures and found the expression pattern of some genes was consistent in both peripheral blood and thymic tissues in MG patients, such as CXCL13 [4, 5], CXCR5 [4], CXCR3 [6], ESR1 [7], IL-6, and RANTES [8].

The current management of MG includes acetylcholinesterase inhibitors, immunomodulatory agents, and thymectomy. Plasma exchange or intravenous immunoglobulins (IVIg) are used for myasthenic crisis [9]. There are none that specifically target the autoimmune deficiency in MG [10]. The category of MG patients is complex. Myasthenia gravis could be classified according to the antibody specificity [acetylcholine receptor (AChR), muscle-specific receptor tyrosine kinase (MuSK), low-density lipoprotein receptor-related protein 4 (LRP4), and seronegative], age at onset (in children; aged less than or more than 50 years), type of course (ocular or generalized), and thymus histology (thymitis, thymoma, and atrophy) [9]. Therefore, we do not consider anti-AChR-positive and anti-AChR-negative patients specifically. The dysregulation of miRNAs has been described in a variety of autoimmune diseases. However, studies addressing miRNAs in MG are limited. Our research relies on pathway enrichment, pathway crosstalk, functional enrichment, and local areas of pathways (LAPs) analyses to elucidate miRNA-gene interactions in the context of pathways relevant to MG. Our results would be more reliable and informative in designating the direction for further miRNA and genes study in MG and offer new potential targets for therapeutic intervention.

Once again, we thank the editor for editorial efforts regarding our paper and giving us an opportunity to clarify the issues. We also thank Dr. Panse for the helpful comments on our work; this may provide great help to our future research.
